# Process evaluation of a tailored nudge intervention to promote appropriate care and treatment of older patients at the end-of-life

**DOI:** 10.1186/s12877-024-04818-4

**Published:** 2024-02-28

**Authors:** Ella L. Bracci, Adrian G. Barnett, Christine Brown, Leonie Callaway, Magnolia Cardona, Hannah E. Carter, Nicholas Graves, Kenneth Hillman, Xing J. Lee, Steven M. McPhail, Ben P. White, Lindy Willmott, Gillian Harvey

**Affiliations:** 1 Caring Futures Institute, College of Nursing and Health Sciences, Flinders University, Adelaide, South Australia Australia; 2 Australian Centre for Health Services Innovation and Centre for Healthcare Transformation, School of Public Health and Social Work, Faculty of Health, Queensland University of Technology, Brisbane, Australia; 3 Faculty of Medicine, University of Queensland, Herston, Queensland Australia; 4 Royal Brisbane and Women’s Hospital, Herston, Queensland Australia; 5 Institute for Evidence-Based Health Care, Bond University, Robina, Queensland Australia; 6 Duke-NUS Postgraduate Medical School, National University of Singapore, Singapore, Singapore; 7 School of Clinical Medicine, UNSW Medicine & Health, Southwest Sydney Clinical Campuses, Discipline of Critical Care, Sydney, NSW Australia; 8 Australia Centre for Heath Law Research, Faculty of Business and Law, Queensland University of Technology, Brisbane, Queensland Australia; 9 School of Psychology, University of Queensland, Herston, Queensland Australia

**Keywords:** Non-beneficial treatment, End-of-life, Clinical audit, Nudge intervention, Hospital

## Abstract

**Background:**

Non-beneficial treatment affects a considerable proportion of older people in hospital, and some will choose to decline invasive treatments when they are approaching the end of their life. The Intervention for Appropriate Care and Treatment (InterACT) intervention was a 12-month stepped wedge randomised controlled trial with an embedded process evaluation in three hospitals in Brisbane, Australia. The aim was to increase appropriate care and treatment decisions for older people at the end-of-life, through implementing a nudge intervention in the form of a prospective feedback loop. However, the trial results indicated that the expected practice change did not occur. The process evaluation aimed to assess implementation using the Consolidated Framework for Implementation Research, identify barriers and enablers to implementation and provide insights into the lack of effect of the InterACT intervention.

**Methods:**

Qualitative data collection involved 38 semi-structured interviews with participating clinicians, members of the executive advisory groups overseeing the intervention at a site level, clinical auditors, and project leads. Online interviews were conducted at two times: implementation onset and completion. Data were coded to the Consolidated Framework for Implementation Research and deductively analysed.

**Results:**

Overall, clinicians felt the premise and clinical reasoning behind InterACT were strong and could improve patient management. However, several prominent barriers affected implementation. These related to the potency of the nudge intervention and its integration into routine clinical practice, clinician beliefs and perceived self-efficacy, and wider contextual factors at the health system level.

**Conclusions:**

An intervention designed to change clinical practice for patients at or near to end-of-life did not have the intended effect. Future interventions targeting this area of care should consider using multi-component strategies that address the identified barriers to implementation and clinician change of practice.

**Trial registration:**

Australia New Zealand Clinical Trial Registry (ANZCTR), ACTRN12619000675123p (approved 06/05/2019).

**Supplementary Information:**

The online version contains supplementary material available at 10.1186/s12877-024-04818-4.

## Strengths and limitations of this study

- Process evaluation embedded within a stepped wedge randomised controlled trial.
- Theory informed process evaluation framed by the Consolidated Framework for Implementation Research (CFIR).
- Qualitative data collected pre- and post-implementation from a range of stakeholders and clinical teams across the three intervention sites.
- COVID-19 may have impacted on recruitment of interviewees and time available to participate in interviews.
- Process evaluation specific to one state in Australia, which may have implications for transferability of findings to other contexts.

## Background

Australia has an ageing population. Older people tend to experience age-related decline including chronic health conditions, multi-morbidity, polypharmacy, and increased levels of frailty which can lead to deterioration and hospitalisation. The current fall-back position for older people with frailty experiencing deterioration is hospitalisation. It is estimated that between 12 to 38% of hospitalised patients are provided with non-beneficial treatments while admitted to hospital at end-of-life [1, 2]. Many of these older people with frailty who are not recognised as being terminally ill by the health system.

The concept and boundaries of non-beneficial treatment, or treatment that is ‘futile’, ‘potentially inappropriate’ or ‘disputed’ are contested [3, 4]. There is some consensus that it would include treatments that have a low chance of achieving a meaningful benefit for a patient in terms of quality and duration of life [4]. There are numerous reasons for providing potentially non-beneficial treatments including failure to recognise these patients as terminally ill, failure to share the knowledge that these patients are near end of life with the patients themselves, patient and family wishes, clinician discomfort with death and dying, clinician workload, perceived legal obligations, and hospital and health system design and culture [5]. In a survey of 349 clinicians, 91% reported providing futile care which led to evasive behaviours such as avoiding end-of-life discussions with a patient or a patient’s family [1]. An Australian multi-centre retrospective cohort study (*n*=831 patients) indicated that two key drivers of non-beneficial treatment were conflict within a family and conflict within the medical team [6]. Other research indicates the provision of non-beneficial treatment relates strongly to the clinician’s experience, beliefs, and their understanding of the concept [1, 7–10]. This is supported by the fact that one third of all emergency calls in hospitals are for end-of-life issues, indicating that it is not until the last few hours or days of life that a patient is recognised as being terminally ill [11].

The financial costs of non-beneficial treatment are substantial. A 2016 study in three Australian hospitals estimated non-beneficial bed days could cost the national health system AUD$153 million annually, which the authors considered a conservative estimate [12]. A recent economic analysis also indicated that up to 24% of overall health expenditure in some countries is attributable to patients in their last three years of life [13]. The provision of non-beneficial treatment can also be harmful to patients, providing potentially burdensome treatment and impacting quality of life. It can also cause moral distress to clinicians [14]. Due to the considerable impact on patients and their family, the economy, and the health care system, reducing non-beneficial treatment is important.

A number of validated tools exist to identify health decline [15]. Two tools used to identify deterioration and mortality in the acute care setting are the Supportive and Palliative Care Indicators Tool (SPICT) [16] and Criteria for Screening and Triaging to Appropriate aLternative Care (CriSTAL) [17]. The SPICT tool aims to identify patients with fluctuations in health status including indicators of health deterioration such as unplanned hospital admission and low muscle mass, and life-limiting illnesses such as cancer, dementia, and frailty to encourage the review of current care and planning for patient centred needs. The CriSTAL screening tool aims to identify end-of-life status of patients on hospital admission. CriSTAL includes seven chronic conditions, recent health service utilisation and acute decompensation parameters as predictors of death for older patients and frailty. With both the SPICT and CriSTAL clinical tools, patients receive a numerical score depending on how many of these indicators are present.

Nudge interventions have been identified as a strategy that can prompt evidence-informed clinical decision making. A recent systematic review identified various approaches to nudging including presenting social benchmarks, providing decision aids, templates to complete upon patient admission, audit and feedback, and modifying default choices [18]. This same review reported nudge interventions have been used to improve the delivery of health care and prompt the review of patient treatment plans, for example, changes to the rate of antibiotic prescribing through audit and feedback alerts where inappropriate prescribing may be present [18].

### The Intervention for Appropriate Care and Treatment (InterACT) intervention

The InterACT intervention involved the implementation of a tailored nudge intervention in three large Australian hospitals in Brisbane. The intervention design was a multi-centre stepped wedge randomised controlled trial with an embedded process evaluation informed by the Consolidated Framework for Implementation Research (CFIR). The stepped-wedge design involves each site being monitored for a usual care phase, followed by implementation of the intervention and subsequent intervention phase. Sites switch over from usual care to intervention sequentially in an order that is randomised before trial commencement. The InterACT protocol [19] and outcome papers [20] are published elsewhere.

Patients aged 75 and over admitted under the care of a participating clinical team were screened bi-weekly (Monday and Thursday) by auditors using the general indicators of the SPICT tool and the CriSTAL tool. Within the intervention phase, patients deemed at-risk of short-term death were reported via a two-fold notification system. The first notification was a real-time alert, while the second notification was an audit report email sent to clinicians caring for at-risk patients at the end of each screening day. The first notification varied across the three hospitals: one hospital used a visual flag displayed on the ward electronic patient journey board; the other two hospitals used an alert that was attached to the patient’s electronic medical record or medical handover report. Clinical teams participating in the intervention represented general medicine, cardiology, stroke, neurosurgery, thoracic medicine, vascular and orthopaedics specialties. These teams had a consistent history of caring for patients aged 75 and over and could tailor their response to the audit report to ensure it was relevant and appropriate to their clinical setting and patient demographic. It was anticipated that increased knowledge of patient risk would trigger a proactive response, such as reducing the time to a clinical review discussion, reducing time to document care review measures (for example, completing an acute resuscitation plan), discussion of the implications of the poor prognosis with the patient, or accelerating a palliative care referral. Within the trial, these three indicators – clinician led review, review of care directive measures, palliative care referrals – were measured as indicators of appropriate care at end-of-life. The published findings of these indicators show that the InterACT intervention did not achieve the intended goal of prompting clinicians as intended [20]. This process evaluation paper aims to provide an explanation for the observed findings that InterACT did not change clinical practice to improve appropriate care at end-of-life.

### InterACT implementation process

The InterACT intervention included both core (fixed) and adaptable (flexible) components at the pre-implementation, implementation, and post-implementation phases (supplementary Table 1). The core components were the assembly of a site-specific executive advisory group and hospital study team made up of a site lead, clinical auditors undertaking twice weekly audits of patient records, and the study research team. Adaptable components related to the tailoring of the alert once patients were identified as being at end-of-life, the specific clinical response, the number of participating clinical teams per hospital and the clinical specialities represented (Table S1). A logic model is in the supplementary files (Table S2).

The executive advisory group assisted with awareness building, recruiting the clinical teams, developing the feedback loop, and advocating for the InterACT intervention to clinicians where needed. The site coordinator or “champion” was expected to be a senior staff member (nurse or alternative) with a solid knowledge of each hospital’s system. They also had a role in awareness building, supporting local context assessment and clinical team participation and tailoring the feedback response.

Clinical auditors at each site were senior registered nurses, employed by the hospital and allocated specifically to the trial to be trained in the use of screening tools and the intervention database. The intervention research team consisted of a group of clinical and context experts, analysts, an intervention coordinator and project manager who were responsible for recruitment of participants and site study staff and conduct of the trial.

### Aims

We present the process evaluation undertaken alongside the main effectiveness clinical trial to report how implementation progressed in each of the three hospitals and provide insights into why the intervention did not have the intended effect on clinical practice related to appropriate care at end-of-life.

The aims of the process evaluation were to:

1. Assess the implementation of InterACT through the lens of a commonly used implementation framework, the Consolidated Framework for Implementation Research (CFIR).
2. Identify contextual barriers and enablers of implementation within and across the three participating hospitals.
3. Provide insights into the lack of effect of the InterACT intervention on appropriate care at end-of-life.

## Methods

The process evaluation was conducted using a descriptive qualitative approach, guided by CFIR. Hospital names are not reported and referred to as: Hospital X, Y and Z to avoid identification.

### Consolidated Framework for Implementation Research (CFIR)

The CFIR framework comprises five constructs or domains that influence implementation processes and outcomes, namely: characteristics of the intervention; the individuals involved in implementation; the inner context; the outer context; the implementation process employed [21]. These five constructs framed the data collection and analysis.

### Process implementation data collection and analysis

Data for this process evaluation were collected via semi-structured interviews with stakeholders at the participating hospitals. These included clinical team leads, members of the Executive Advisory Group, the clinical champion/site lead for the intervention, auditors and research project team members who interacted directly with the hospital staff. Approximately 20-23 interviews across the three sites were expected to be conducted with the stakeholders listed above.

Interviewees were recruited via convenience purposive sampling by research project team members who had on-site contact with the hospital stakeholders. If interviewees agreed to be involved in the process evaluation, written consent was obtained, and their details were forwarded to qualitative researchers GH (PHD) and EB (MS) to schedule and conduct the interview. The interviewers were not involved in delivery of the trial or on-site implementation and therefore not known to interviewees. Interviews were completed at two time points: during the first stages of InterACT implementation (November 2020 to March 2021), and once the intervention was completed (June to July 2021). Interviewees who participated in the first round of interviews were re-contacted and invited to the second round of interviews. For interviewees who were unable to be re-interviewed in the second round, alternative team members and clinicians were nominated. Only the interviewees and interviewer were present at the time of interviews.

Interviews were in-depth and semi-structured with prompts informed by the CFIR constructs [21] (supplementary S6). The interviews aimed to explore clinicians’ views on the InterACT intervention, how clinical teams used the feedback provided, and any barriers or enablers to the implementation process. Interviews were conducted either over the phone and digitally audio recorded or using Zoom (Video Communications Inc.) with live audio transcription. One researcher (EB) coded the data from qualitative interviews to the CFIR framework in NVivo software (Release 1.0, Version 12, QSR International) and discussed the initial analysis with a second researcher (GH) to verify the coding. Data that did not map to the CFIR constructs was inductively analysed and cross-checked by two research members until agreement was achieved. Interview participants were not provided with a returned copy of their transcript for comment or correction, nor were they approached for feedback on the findings.

## Results

In total, 20 pre- and 18 post-intervention interviews were conducted across the three hospitals (Table 1). Of the 18 post-intervention interviews, 14 were repeat interviews. Hospital X had the largest number of interviews (*n*=16) compared to Hospital Y (*n*=10) and Hospital Z (*n*=12). Interviews were an average of 16.2 minutes (range 10.5-26.8) in Hospital X, 13.5 minutes (range 3.4-18.5) in Hospital Y and 17.1 minutes (range 11.1-27.1) in Hospital Z.

**Table 1:** Overview of interviewee’s role and pre and post-intervention interviews across hospitals

	**Preliminary Interviews (*n*=20)**	**Post intervention Interviews (*n*=18)**
**Hospital X**		
Clinician	5	6
Executive Advisory Group	1	0
Auditors	2	2
**Hospital Y**		
Clinician	3	2
Executive Advisory Group	2	1
Auditors	1	1
**Hospital Z**		
Clinician	3	3
Executive Advisory Group	1	3
Auditors	2	0

Clinical teams across hospitals include cardiology, stroke, general medicine, palliative care, respiratory, geriatrics, thoracic, orthopaedics and emergency

### Findings

We begin by presenting each hospital as a case, detailing findings related to the CFIR constructs, namely: perceptions of the InterACT intervention; individual beliefs about care at end-of-life and motivation to change; contextual barriers influencing implementation; and the implementation process. Detailed mapping to CFIR is in supplementary tables S3, S4 and S5. We then summarise the key process evaluation findings at an overall level (Table 1).

### Hospital X

Hospital X clinical teams opted to tailor the InterACT intervention by the introduction of a visible orange flag next to the patient’s name if they were identified as ‘at risk’ following the first notification. The orange flag was displayed on the Wardview, an electronic patient journey board in each hospital ward. Auditing was completed using mostly paper-based records. This often meant that auditors were required to find physical notes on the hospital ward which could require multiple visits and took longer overall to collect the required information.

The high visibility of the feedback loop was identified as a key component in activating a clinical response and served as a useful prompt to clinicians compared to the scheduled emails that were sent to clinical leads from the auditors.

*“Every time that happens (presence of orange flag), I go, oh yes, I’m supposed to be doing this”* [Clinician 3]

Though clinicians felt the premise of InterACT was beneficial and aligned with their stance on non-beneficial treatment, some felt they were already proficient at identifying patients at or near the end-of-life or questioned the over sensitivity of the SPICT screening tool. Other clinicians commented that seeing a high number of patients flagged was consistent with the demographic of patients presenting to the hospital.

Different clinicians reported differing experiences with identifying patients at or near end-of-life and initiating palliative care discussions. Some reported fears around ceasing treatment or taking a different approach as this could feel like they were failing their patients by not providing treatments and investigations.

*“They’re (clinicians) fearful of any discussion and stopping something or not doing, I think they’re worried they’re going to get blamed or have their registration challenged or something*” [Clinician 6]

Additionally, there were concerns that the practice change required for InterACT could create additional workload. This was problematic given the increased pressure during the COVID-19 pandemic and the expectation to work harder and faster due to the increasingly constrained public health budget as well as the demand for services and the increasing costs of healthcare delivery.

*“People are so busy and running around like headless chooks*^1^* [chicken]. It's not front of mind. I don't think it's going to be an easy process to get people to change the way they do things”* [Clinician 3]

Although prior to InterACT implementation there were issues with governance, obtaining relevant agreements and convening the Executive Advisory Group, there was good support from this higher level which facilitated confidence within clinical teams and assisted with implementation. The executive advisory group was seen to be well-functioning, although still experienced issues identifying and attending meetings at convenient times, resulting in members being unable to attend and contribute to the discussions.

*“Critical (Executive Advisory Group) to the success of getting it (InterACT) up and going, because I think sometimes when you don’t have the support to facilitate and negotiate, things sometimes tend to derail”* [Executive Advisory Group 1]

Initial awareness of InterACT amongst participating clinical teams was strong and well received following an education session from the research project team. However, the COVID-19 pandemic resulted in an 8-week delay between clinical team recruitment and trial commencement, resulting in some loss of momentum. Clinicians felt that having a face-to-face refresher meeting could re-engage clinical teams, ensure awareness was ongoing and prevent project fatigue. Meetings over Microsoft Teams were considered challenging with minimal engagement and contributions from attendees. As an alternative to a refresher meeting, one clinician suggested that having an onsite InterACT project officer could facilitate greater engagement from clinical teams and increase awareness, for example, by prompting clinicians and staff to the orange flag and encouraging discussion about appropriate treatment.

At a wider contextual level, palliative care clinicians reported that local policy changes resulting in a move from a community-based model to a hospital focused model of palliative care had resulted in higher numbers of admissions and bed pressures. Consequently, clinicians felt it would be difficult to distinguish the effects of the InterACT intervention from wider changes in palliative care at the hospital and community level.

*“It was really quite odd that we had to report to a community director when we were a ward on a hospital campus. We've been put under a different type of pressure now more about the mechanics of admissions to our ward as in if we've got empty beds, that's a bad thing we have to fill with anybody, everybody. It's hard to now pick whether this study, looking at a different phase of life, has led to changes there, if anything, we seem to get more referrals”* [Clinician 6]

### Hospital Y

The initial InterACT intervention feedback loop at Hospital Y was tailored to be a referral prompt in the electronic hospital system attached to the medical handover report. This notification was intended to be displayed on the medical handover form used by participating clinical teams during each ward round. The medical records were mostly paper based with some electronic information available. The findings in Hospital Y demonstrate greater variability in terms of views about the intervention and clinician responses to InterACT. For some clinicians, flagging of patients was not perceived to provide an advantage over existing practice because they believed they could already identify at risk patients as part of their routine clinical practice.

*“But we generally had identified our patients so the InterACT study criteria didn't necessarily achieve anything different out of our patient cohort”* [Clinician 7]

Other clinicians did not accept the evidence underpinning the intervention, with some viewing it as ageist.

*“One of their cardiologists came back and said they didn't like the science around this and disputed a lot of it*” [Executive Advisory Group 3]

Conversely, some clinicians perceived the quality and validity of evidence supporting InterACT as highly credible. In some cases, the relevance of the screening tools to some patient groups was questioned as they evaluate a patient’s prior level of function. As such they were viewed less helpful for patients admitted after an acute episode such as stroke, where it can be difficult to make definitive predictions in the early days after admission.

Overall, leadership engagement and commitment were important in supporting and driving implementation and getting clinical teams enrolled in InterACT. Some interviewees perceived that the current handling of end-of-life discussions required significant change indicating a likely readiness and tension for change.

*“It has been a real eye opener that you’ve got all these red flags and no one’s having these conversations with these people, it’s like why? Why are people not even referring them or doing something”* [Auditor 3]

Advance care planning was referred to as ‘undervalued’ by some interviewees who highlighted the importance of shared decision making with patients about their health journey as opposed to a biomedical cure all approach which was prevalent with some clinicians.

*“You’re not dead until you’ve had your sixth bone marrow transplant and your 14*^*th*^* trip to the intensive care. That kind of hope at all costs with every possible intervention”* [Executive Advisory Group 3]

Although stakeholders were initially on board in the planning and engagement stages, by the time the intervention was ready to be implemented, some of the key people were no longer involved causing issues related to budget and in-kind agreements. There were also problems nominating a clinical site lead to act as a champion for the InterACT intervention. A lack of commitment from senior medical staff resulted in the identification of a site lead who was not as well known or situated within the organisation.

Like Hospital X, Hospital Y had an 8-week project suspension due to the COVID-19 pandemic which may have impacted implementation and momentum. The time delay, combined with in-house logistical issues, affected the recruitment and appointment of auditors, which in turn led to instability in the initial audit team. Auditors also highlighted issues with documentation and a lack of detail in medical notes which added complexity to the screening process. For example, differing levels of detail in Acute Resuscitation Plans made it difficult for auditors to determine if a conversation had taken place between the clinician and the patient and or family.

The auditing process was further complicated by a partial electronic medical record system. The patient notes were paper based whilst the patient was in hospital, then scanned and uploaded into the electronic system after discharge. As auditing was undertaken prospectively, not all the required information could be located electronically. This meant that the auditors had to visit the ward to access paper notes, which sometimes caused confusion with medical staff and competition for patient charts.

### Hospital Z

Hospital Z was the last to switch over into the intervention phase and, as such, had the shortest intervention period. Hospital Z had fully electronic medical records, which meant that all the auditing could be completed with digital patient records. Participating clinical teams elected to tailor the initial InterACT feedback loop to be an alert in a patient’s medical record.

Key stakeholders reported positive perceptions regarding the InterACT intervention, referring to it as a recognisable area for clinical practice improvement. Clinicians appeared to value the evidence underpinning InterACT and felt that it could address some of the barriers to non-beneficial treatment. Clinicians mostly reported that the screening tools were appropriate in identifying patients at risk or close to end-of-life. Despite the support for InterACT, the alert in the patient’s medical record was seen to lack visibility.

*“Unless they were on ward service and they were seeing these patients, it may not have stayed on their radar”* [Clinician 13]

Clinicians generally expressed positive beliefs and knowledge regarding InterACT, referring to the intervention as both relevant and beneficial in prompting the review of clinical management and care for older patients. This was particularly the case for teams whose patients tended to have chronic conditions and longer hospital stays. Though there were positive views regarding InterACT, clinicians acknowledged that practice change could take time and be difficult to sustain without ongoing support.

*“Culture change requires years, and it requires resourcing to put the culture change in and then once it’s there, the hardest thing is to keep the change there”* [Executive Advisory Group 5]

Leadership engagement and support for InterACT was high in Hospital Z, although there were some issues engaging clinical teams to take part in the intervention, particularly the larger clinical teams. Differences of opinion were apparent between the clinicians interviewed with regards to conversations about end-of-life. Some were seen to favour a more biomedical or ‘death denying’ approach rather than engaging with patients and families to understand their needs at or near end-of-life.

*“I was never trained to do this, I don’t have the expertise to do this, I don’t think it’s my job because my job as a doctor is to cure and to death deny”* [Executive Advisory 5]

However, other clinicians were committed to early conversations about palliative care with patients and family members, reporting that their patients welcomed such conversations.

Staff in Hospital Z showed a general willingness to engage in research and form external partnerships. While one clinician felt that there was no “push anywhere else in the system” regarding non-beneficial treatment, the recent move towards assisted dying legislation in Queensland was seen to be raising awareness about end-of-life matters.

*“End-of-life care, palliative care, advanced care planning are not funded appropriately and it’s very clear in Queensland”* [Executive Advisory Group 5]

Some clinicians reported feeling that they lacked training and were ill-equipped with regards to goals of care communication and advanced care planning. When patients moved between clinical teams within the hospital this created uncertainty about which clinical team had responsibility to initiate end-of-life conversations.

Another issue raised by interviewees related to state-level legislation that affected the delivery of care to patients who lacked decision-making capacity. In the context of InterACT, this could mean that relatives and loved ones of patients might choose to continue treatments and investigations that could be considered non-beneficial, and clinicians felt compelled to provide non-beneficial treatment due to legal concerns. This demonstrates the potential for legislation to have unintended consequences related to non-beneficial treatment.

*“Sometimes relatives just don’t want us to give up, even though we know the patient is dying, and sometimes we are compelled to do more because relatives have a lot of power here in Queensland because of the legislation”* [Clinician 14]

COVID-19 resulted in delays to trial commencement. The delays also caused difficulties in identifying key personnel and site leads, and in creating momentum at the initial stages. Despite some initial complexities, clinicians and key stakeholders felt well prepared for InterACT with good support from the research team. Clinicians highlighted the key role of the research partners in ensuring successful implementation and reported satisfaction with communication about the intervention.

The auditing process was relatively straightforward using the digital record system, which meant that auditors could extract information from the patients’ notes in a relatively short time, although documentation about end-of-life discussions and acute resuscitation plans were lacking in detail at times.

### Summary of key findings across hospitals

Detailed quotes mapped to each of the CFIR domains for each hospital are presented in the Supplementary files (Tables S3, S4 and S5) while a summary is presented in Table 2. The three hospitals faced similar issues concerning the timeliness and usefulness of the email notifications for at risk patients, perceived sensitivity of the screening tools and barriers relating to clinician beliefs and self-efficacy regarding non-beneficial treatment and end-of-life care (Table 2). Strong leadership engagement and support from the Executive Advisory Group and research team were implementation facilitators. Inductive analysis of data that did not map to CFIR indicated some concern with the future sustainability of InterACT and transferability to other cohorts. Further themes included the need for economic analyses to determine cost-effectiveness and the importance of pre-existing partnerships and collaborations. (Table 2, Tables S3, S4, S5).

**Table 2:** Summary of Key findings across InterACT hospitals in relation to CFIR constructs

**CFIR Construct**	**Hospital X**	**Hospital Y**	**Hospital Z**
**Intervention**	- Longest time intervention period - Visible alert flag - Fit with existing views about non-beneficial treatment, although concerned about additional workload it could create - SPICT screening tool was seen to be too sensitive by certain clinical teams	- Document containing alert was not used by most clinical teams - Limited perceived advantage of InterACT – clinicians felt they were already able to identify end-of-life patients - Mixed views about the strength of evidence underpinning InterACT and relevance of screening tools	- Shortest intervention period - Poor visibility of response/alert - Screening tools were seen to be appropriate - Difficulty engaging clinical teams
**Individuals**	Some clinicians: - Believed they were already proficient at identifying individuals at end-of-life - Questioned sensitivity of the screening tools - Expressed a fear of failing patients if treatment ceased	- Mixed views amongst clinicians about the need for change - Difficulty identifying a site champion	- Differences of opinion amongst clinicians about end-of-life care - ‘Death-denying’ views present amongst some - Perceived lack of educational preparation in end-of-life care - Limited dissemination of audit feedback data beyond the senior clinician
**Inner Setting**	- Strong support from the Executive Advisory Group	- Initially had strong senior leadership for the project, but change of staff resulted in reduced engagement of Executive Advisory Group - Loss of Advance Care Planning facilitator and lack of clarity about who was responsible for Advance Care Planning	- Strong leadership engagement and support - Organisational culture supportive of research
**Outer Setting**	- COVID-19 delays to implementation affected the momentum - Influence of wider policy changes on delivery of palliative care - New quality standards around palliative care	- COVID-19 delays	- COVID-19 delays Enactment of assisted dying legislation increasing awareness of end-of-life issues - Influence of existing policy and legislation
**Implementation Process**	- Paper record system meant auditing took longer, however, visibility of auditors on the wards increased awareness of the intervention - Educational preparation from the research team was good, although may have benefited from refresher sessions - Potential benefit of having an on-site project facilitator	- Support from research team was perceived to be good, although opportunities to raise awareness further were identified, - Challenges in the auditing process	- Well prepared and supported by the research team - Auditing process relatively quick and efficient with electronic record system
**Other**	- Awareness building - Role of other health professionals - Value of research opportunities - Sustainability and other cohorts	- Value of pre-existing partnerships - Transferability to other cohorts	- Economic research impact - Sustainability

## Discussion

This process evaluation assessed the implementation of the InterACT intervention. We seek to understand why the intervention did not appear to change or improve clinical practice related to providing appropriate care at end-of-life. The findings illustrate the complex interaction of enablers and barriers that played out during the process of implementation, particularly at a clinical level.

Whilst there was general support for the rationale behind the intervention, namely the need to identify patients near the end-of-life and provide appropriate care, there were clearly barriers that limited changes in practice. In a few cases, the evidence itself was contested, as was the relevance of the tools that were used to screen patients. However, more significant barriers appeared to relate to the response to the nudge intervention and how it was incorporated into routine practice, clinician beliefs about care at end-of-life and perceived self-efficacy to engage in end-of-life discussions, and wider contextual factors in the health system. Each of these is discussed in turn.

### The nudge intervention and incorporation into routine practice

Nudge and feedback loop interventions have previously been successful in healthcare settings [18]. A systematic review of 48 nudge interventions found that over 70% significantly improved clinical decisions [18]. However, the ‘potency’ of the nudge intervention was an important factor, as in some cases the nudge was insufficient to create practice change or influence clinical decision-making. The most successful nudges were those that were part of a multi-component intervention, changed default options, and enabled choice. In contrast, simpler nudge interventions that used reminders to prompt implementation activities did not significantly alter clinical behaviour [18]. In the case of InterACT, the prospective feedback loop to alert treating clinicians to patients requiring end-of-life care and ‘nudge’ an appropriate response may not have been a powerful enough prompt to enact change, particularly given the interacting influence of other barriers to implementation. Further, nudge interventions assume that the system being nudged is operating effectively, which given wider contextual factors, namely, the concurrent COVID-19 pandemic, was often not the case. It is possible that the intervention was nudging a system which, for the factors highlighted in the findings, was not wholly receptive or able to enact the required change. It may be that the nudge needed to be directed at staff, specifically trained and comfortable with conducting honest and empathetic discussions with terminally ill patients and their carer’s.

In addition to the ‘potency’ of a nudge, how it is incorporated into routine practice is an important consideration. As reported in the systematic review, information provision should occur at opportune times to ensure it is ‘top-of-mind’ and prompts the anticipated clinical decision making [18]. In the InterACT intervention, the visibility of the initial alert varied across the three hospitals. Incorporating the alert into the ward-based electronic patient journey board in Hospital X made it highly visible and was an important enabling factor. More generally, the first stage of creating an alert appeared more useful to clinicians than the circulation of the email reminders twice weekly. This timing did not always align with patient throughput on the participating wards and some of the clinicians nominated to receive the emails complained of email overload, which impacted their response.

### Clinician beliefs and perceived self-efficacy

Barriers at the clinical level appeared significant and pivotal to the response to the InterACT intervention. Whilst many of the clinical staff agreed that identifying patients who are terminally ill was important and that there was room for improvement in this area of care, the shift towards palliation and away from a biomedical “cure all” approach is clearly challenging and requires major cultural change. Differing views and beliefs about reducing treatment at end-of-life were apparent, as were perceptions of self-efficacy to engage in end-of-life discussions with patients and family members. Much of the feedback from the treating clinicians included them questioning their role in the end-of-life discussions and emphasized their training was more conventional interventions aimed at short-term improvement.

The InterACT findings mirror barriers reported in the wider literature on providing end-of-life care, including, a lack of education and training, difficulty in prognostication, and cultural differences. For example, a cross-sectional survey conducted in the United States looking at preparation of staff to provide optimal end-of-life care indicated that a third of medical and surgical practitioners experienced uncertainty in knowing when to refer patients to hospice and palliative care services, and forty per cent felt unprepared to deal with patients and family members [7]. Low self-efficacy to manage end-of-life care, coupled with pressure from patient’s families, was seen to contribute to the provision of futile treatment [7]. Poor clinician understanding about older patients’ or family readiness to discuss end-of-life prognosis and management can contribute to a lack of action following identification that a patient is at risk of death, as reported in an Australian survey of clinicians and members of the public [22].

An Australian three-centre retrospective cohort study of 831 patients found that up to 12% were receiving non-beneficial treatments [6]. The two main drivers of non-beneficial treatment were conflict within a family and conflict related to the medical team [6]. A separate Australian study involving semi-structured interviews with 96 doctors in specialties providing end-of-life care identified a range of causes of futile or non-beneficial treatment, in particular the influence of family or patient requests and doctors being locked into a curative role [5]. Other international studies, for example, research conducted in the Netherlands [9] and the United States [1], suggest that patient and family members can create conflict for clinicians by requesting treatments that are considered futile. In the cross-sectional survey of medical and nursing clinicians at two academic hospitals in New York [1], over 90 per cent of respondents indicated that they had provided, or possibly provided, futile or potentially inappropriate care in the preceding six months. The main reasons cited for providing futile care were requests from the family of patients (61%), or a physician or consultant request (11%) [1]. This concurs with some of the findings from the process evaluation, particularly concerning legislative influences on practice in the Australian state where the study was conducted. Pressure from family could have been related to the way clinicians conducted the narrative, emphasising the possibility of active intervention rather than an honest discussion around the possibility of the patient being terminally ill.

### Wider contextual factors

Alongside legislative influences noted above, other contextual factors presented barriers or enablers of implementation. The Executive Advisory Groups generally functioned in an enabling way, despite difficulties in assembling and meeting at mutually favourable times, as they provided explicit and visible support for the project. Internally appointed site leads were also intended to function as champions and act as an enabler of implementation. Champions with formal authority who are well-respected by colleagues can help to launch implementation efforts and promote responsiveness amongst their peers [23]. Important champion attributes include influence, enthusiasm, physical presence, persuasiveness, grit, and participative leadership style, coupled with skills in negotiation skills and communication [24]. Absence of site champions with the requisite attributes or level of influence presented a barrier to implementation as there was some difficulty identifying and retaining key personnel and site leads across the three hospitals. At one hospital the site lead was not particularly well known, and this lack of a recognised clinical champion could have reduced staff buy in to InterACT. In turn, reluctance amongst clinicians to volunteer for the site champion role could relate to the timing of the intervention taking place during the COVID-19 pandemic, which created higher workloads and pressure on clinicians.

Reflecting on the implementation barriers and enablers and the insights the findings provide about why the InterACT intervention did not change practice relating to end-of-life care, several implications for future initiatives to improve end-of-life care can be identified. In terms of intervention design, adaptations and enhancements to the nudge intervention would be warranted. These could include developing screening mechanisms that do not rely on a standalone auditing process conducted by individuals external to the clinical practice setting. Developing more automated, real-time, and visible flagging of patients at end-of-life would be advantageous and likely plausible in the near-term with ongoing advancements in digital health care systems.

However, over and above improvements to the nudge intervention, a stronger focus on activating a response to the resultant flags is critical. This needs to take account of clinician-related beliefs and self-efficacy to care for patients and families at end-of-life. This would likely require incorporation of a nudge intervention within a multi-faceted implementation strategy that addresses cultural barriers and encompasses clinician education to build capability and confidence. This could be further enhanced by providing feedback on performance over time, thus creating a more active, as opposed to passive, audit process. As part of developing multi-component implementation strategies, it is also important to build on identified enablers such as executive support and sponsorship and a designated opinion leader or clinical champion role. Although likely to be more time and resource-intensive, evidence supports the need for this type of multi-faceted approach when complex change strategies are required to address multiple and multi-level implementation barriers [25] something that a future research agenda could usefully address.

Much of the research in the terminally ill older people with frailty overlooks or underestimates interventions which empower patients in a genuine way with information about the likelihood that they are terminally ill with the inherent nature of uncertainty that accompanies every medical prognosis. This usually occurs with other terminally ill conditions such as cancer or neuromuscular disease, empowering the patient and carer to decide their own goals of care in the light of the prognosis. As older people with frailty who are terminally ill are treated differently than patients with terminal conditions such as cancer, complex and time-consuming decisions around end of life are left to a system of hospital doctors who may not necessarily be trained to carry out these conversations.

### Strengths and limitations

The process evaluation focused on understanding barriers and enablers to implementation through the lens of the Consolidated Framework for Implementation Research. The qualitative interviews provided rich data; however, recruitment of interview participants was challenging. The interviews with key stakeholders including the executive advisory group, auditors, and clinicians were conducted at a mutually beneficial time. However, due to the interviewee’s busy schedules, in part influenced by the demands of the COVID-19 pandemic, these interviews were often terminated early, rescheduled multiple times, or declined. As a result, there were fewer interviews than anticipated and not all the pre- and post-implementation interviews were conducted with the same individual. Whilst there were many commonalities across the three hospitals, having a broader range of clinical team members interviewed may have been useful to understand the detailed processes of implementation within and across hospitals. Additionally, this research is limited by geographical location and may not be transferable to other states within Australia, to the international context and to other patient cohorts.

## Conclusions

This process evaluation provides important implications and considerations for future interventions aiming to provide appropriate care for older people at the end-of-life. The findings suggest that clinicians generally support the ideas underpinning InterACT to ensure that older people close to end-of-life have their treatment preferences known and acted upon. However, the cultural context, leadership support and implementation readability were insufficient to achieve success. Some clinicians appear to adopt a biomedical approach which may be related to a lack of clinician self-efficacy in end-of-life care discussions, or concerns with potential legal liability. Practice change in this complex area of healthcare is likely to take a considerable amount of time and effort despite positive attitudes towards improving care. It may require more complex, multi-component interventions such as referral to specifically trained staff to address the identified barriers to implementation. Lastly, these findings demonstrate both the need to undertake steps in the pre-implementation phase i.e., context mapping or environmental scans, to identify barriers to implementation, and the need to tailor to local contexts.

### Supplementary Information

**Supplementary Material 1.**

## Data Availability

The datasets used and analysed during the current study available from the corresponding author on reasonable request.

## Abbreviations

ANZCTR: Australian and New Zealand Clinical Trial Registry
CFIR: Consolidated Framework for Implementation Research
CriSTAL: Criteria for Screening and Triaging to Appropriate aLternative Care
HREC: Human Research Ethics Committee
InterACT: Intervention for Appropriate Care and Treatment
QUT: Queensland University of Technology
SPICT: Supportive and Palliative Care Indicators Tool

## Footnotes

1 “Headless chook or chicken” is a slang term used in Australia to describe being highly busy and frantic at times without calmness or higher order planning
