# Supplementary material for: Process evaluation of a tailored nudge intervention to promote appropriate care and treatment of older patients at the end-of-life
Source: BMC Geriatr. 2024 Feb 28;24:202. doi: 10.1186/s12877-024-04818-4 (PMC10900675; doi:10.1186/s12877-024-04818-4)
Supplement: Supplementary file 1 — Supplementary Material 1. [file 12877_2024_4818_MOESM1_ESM.docx]

# **Supplementary Files**

**Table S1.** Summary of key core and adaptable components of InterACT

| **Component** | **Core** | **Adaptable** |
| --- | --- | --- |
| Executive Advisory group | Members:   - CEO/Nominee - Clinical Director Medicine & Surgery - Palliative Care nursing and medical lead - Quality and safety program lead - Consumer representative | 6 to 10 members |
| Hospital Study team | Members:   - Nursing representative - Palliative Care and or Advance Care Planning Representative - 2to 3 clinical auditors - Site coordinator | Shared position |
| Clinical Teams | Determine which clinical specialities have a higher likelihood of patients at risk of NBT through priority sampling and enrol up to 7 clinical teams per hospital. | Combination of clinical specialities could be enrolled |
| Auditing | Screening recently admitted patients using SPICT and CriSTAL to determine risk of short-term death. | Timing** – patient review dates either Monday, Thursday, Friday OR Monday, Tuesday and Friday |
|  | Threshold of 6 for the CriSTAL tool and/or SPICT of 2 | Threshold of SPICT changed to 3 in Hospital X |
| Information and education sessions for clinicians | Content of sessions includes reasons for NBT, frequency, duration and costs associated with NBT, legal and ethical positions, options, review of audit tools. | Timing****** and format i.e., alignment with grand rounds, MDT meetings, clinical updates |
| Feedback loop | Method of ‘flagging’ patients at risk and prompting clinical teams dependent on hospitals systems and IT. | Alert on electronic patient journey board displayed in wards, alert on patient's electronic medical record, alert on medical handover report |
| Feedback mechanism | Dissemination of patient names to at least one senior member of each clinical team | Mode of delivery** i.e., text, email, phone call |
| Clinical Response | Expected to respond to patient being at risk through chosen clinical response | Vary according to type of clinical team and capacity for response i.e., advance care directive, care review, palliative care referral |
| Evaluation | Members of clinical teams, EAG and auditors asked to complete interviews | Number of clinical team members |

MDT = multidisciplinary team; NBT = non-beneficial treatment; EMR = electronic medical record; SPICT = Supportive and Palliative Care Indicators Tool; CriSTAL = Criteria for Screening and Triaging to Appropriate aLternative Care (CriSTAL)

**Though clinicians had a choice of both audit timing and mode of delivery, all clinical teams across the three hospitals selected the same timing of audit and patient review and mode of delivery i.e., email

**Table S2.** InterACT intervention Logic model

**EXTERNAL FACTORS**

Economic imperatives in health care delivery

Challenges in determining beneficence

Legal and clinical practice issues around the end-of-life

Patient, family, clinician and hospital factors

**ASSUMPTIONS**

Clinical teams will benefit from and respond positively to participation.

Clinical teams will provide a clinical response to patient risk profile information.

Site study teams will function well and actively support engagement and participation

Hospitals, via their advisory group, will be active partners and value and sustain participation

| **Inputs/resources** | **Activities** | **Outputs** | **Short-term outcomes** | **Medium-term outcomes** | **Impact** |
| --- | --- | --- | --- | --- | --- |
| Partnership grant and partners  QUT-based project team  Site study team and executive advisory groups  Project plan  Study protocols, data collection tools  Ethical and governance approvals and agreements  Stakeholder engagement | Establish site teams and advisory groups  Review site context and tailor the intervention  Site-specific implementation plan and delivery  Collect data to identify patient risk profiles and complete feedback loops with clinical teams  Complete each phase of the study as per the protocol | 3 complete data sets from 14 clinical teams in three hospitals  Understanding of impact of context, and enablers and barriers in responding to identified risk of non-beneficial treatment at the end-of life. | Change to clinical practice stimulated by receiving objective data  Reduced incidence of patients receiving non-beneficial treatments at the end-of-life  Improved resource use in acute end-of-life care | Evidence-informed changes to care delivery for patient groups at high risk of receiving non-beneficial treatment  Sustained clinician engagement in reducing non-beneficial treatments in acute hospitals | Improved end-of-life care in acute hospitals  New guidelines and policies about end-of-life care for elderly populations  Evidence- informed decision-making re resource allocation in acute end-of-life care |

**Table S3.** Summary of Hospital X interview data using the Consolidated Framework for Implementation Research (CFIR)

| **CFIR domain** | **Construct** | **Quote/Findings** |
| --- | --- | --- |
| **Intervention Characteristics** |  |  |
|  | Complexity | Anything that is more work is harder for people to implement [Clinician 5] |
|  |  | Everything is too busy, too hard, InterACT is the last thing we want to talk about [Research Team 2] |
|  |  | People are so busy and running around headless chook. It's not front of mind. I don't think it's going to be an easy process to get people to change the way they do things. Yeah change is always hard as it is [Clinician 3] |
|  | Design Quality and packaging | It definitely helps to highlight the more vulnerable ones [Auditor 1] |
|  |  | What it has taught us, is to really get on to it faster and to actually really make a definitive move [Clinician 2] |
|  |  | Could have been reinforced some more at various timepoints, just the ensure that the intervention wasn’t stepping down [Clinician 2] |
|  |  | You just start forgetting about it, you just don’t notice it anymore because it’s just there in the background [Clinician 3] |
|  |  | Because we're not an electronic database. So, we have to literally hunt around the ward for charts [Auditor 1] |
|  | Adaptability | They changed the rules on SPICT. They added an extra point [Clinician 3] |
| **Inner Setting** |  |  |
|  | Tension for change | There is a growing recognition that this is something that’s not an all or nothing, but a stop and think question. It might be okay to go ahead with all your exact plans but are you aware that the patient is already at risk potentially of dying in the next 12 months or so? Does that change what you want to do? [Clinician 6] |
|  | Available resources | In the acute setting, it can be difficult to implement [Clinician 2] |
|  | Culture | There’s still this idea amongst some clinicians that you have to do everything at all costs [Clinician 5] |
|  | Relative priority | We are happy to amend certain processes or bring in new ideas, given that there’s a rationale for it [Auditor 2] |
|  |  | Patients who have come through the emergency department and have had three admissions in the last few months and it (ACP/ARP) hasn’t been discussed. I think we can do things better [Auditor 2] |
|  |  | It has helped me focus more on patients that I might have neglected somewhat [Clinician 4] |
|  | Compatibility | It is very easy to become SPICT positive, especially if you have an unplanned hospital admission which all the general medicine patients are [Clinician 1] |
|  |  | We still have a problem in our world of palliative care, we still have a lot of people that don’t do our job and think we do no tests on people, that we do no investigations and that can be really hard, or that they shouldn’t refer someone to us who is still walking and talking [Clinician 6] |
|  |  | We’re being made to admit many more people now, in the past we would have just said this person’s dying in the next few hours, let them stay where they are [Clinician 6] |
|  |  | We don’t use the specific tools you’ve used, we do use things like clinical frailty scores, dementia, but also clinical judgement [Clinician 1] |
|  |  | It’s not out of the ordinary. It is consistent with our approaches [Clinician 2] |
|  |  | I'm a geriatrician in a hip fracture unit, this is our standard sort of bread and butter that we do. All patients get, you know, acute resuscitation plans or wishes and we sort of talk to family, so we do that as a routine practice [Clinician 1] |
|  |  | There’s a lot of loyalty because a lot of these patients have been known to teams for decades and they do honestly feel like they’re letting the down sometimes [Clinician 5] |
|  |  | From a palcare world it was like the answer to our dreams because we need to be empowering other teams to be identifying patients and at a timely, you know not the last three days of life [Clinician 5] |
|  | Implementation climate | Very open to innovative ideas and improvements to directly affect the patients and also for our practices [Auditor 2] |
| **Characteristics of individuals** |  |  |
|  | Self-efficacy | Discussing end-of-life and palliative care is an area that is lacking and now that I’m a part of this project is something that’s objective [Auditor 2] |
|  |  | I haven’t been quite as good as I could in initiating those discussions earlier on [Clinician 4] |
|  |  | Every time that happens (presence of orange flag), I go, oh yes, I’m supposed to be doing this. It’s not part of my inner being yet [Clinician 3] |
|  |  | Weren’t (clinical teams) as aware as I thought they would be around the documents, around the general principle of advanced care planning [Clinician 5] |
|  | Knowledge and information | Fearful of any discussion and stopping something or not doing, I think they’re worried they’re going to get blamed or have their registration challenged or something [Clinician 6] |
| **Implementation Process** |  |  |
|  | Engaging key stakeholders | Very cohesive, very positive advisory group and very good interactions and easy interactions with each of the clinical teams [Research Team 2] |
|  |  | Critical (EAG) to the success of getting it (the project) up and going, because I think sometimes when you don’t have the support to facilitate and negotiate, things sometimes tend to derail [Executive Advisory Group 1] |
|  |  | There were a whole bunch of times where it (EAG) met and I didn’t even get to it because I was either in another meeting or not here, just the meeting times were not good [Clinician 3] |
|  |  | Getting that type of group together is really challenging and they just want the facts and the numbers [Clinician 5] |
|  | Opinion leaders | I’ve been on the advisory board and with the team, which has been really successful, I think that's enabled us to understand any barriers or any anything that is put precluding us from progressing. I've sort of offered myself up and from time to time being the escalation point between meetings, if you like, if there's been a concern [Executive Advisory Group 1] |
|  |  | It had executive sponsorship from the very beginning and so that that was there in place. And obviously our department is also very research active so we're keen for that [Clinician 2] |
|  | External change agent | QUT team has been exceptional. They’ve been great. It’s been a pleasure [Executive Advisory Group 1] |
|  |  | Very well conducted, you guys kept us in the loop all the time, just shows the calibre or the people behind it [Clinician 4] |
|  | Planning | It was done really carefully, it was done over quite a long time, it was something that came with good warning, good preparation [Clinician 6] |
|  |  | I think if we had a better understanding of what information we wanted, perhaps it would have been a bit easier for us to arrange our time, I was sort of trial and error because we weren’t really sure how any charts we were going to get [Auditor 2] |
|  |  | If the paper record is not available, if someone else is using it, you can't look at it. It's just that slows them down greatly [Auditor 1] |
| **Outer Setting** |  |  |
|  | External Policy and incentives | It’s hard to now pick whether this study, looking at a different phase of life, has led to changes there, if anything we seem to get more referrals now, really, really, imminently dying people, but with pressure to take over care rather than just give consultant advice [Clinician 6] |
|  |  | Jumped on the back of these new standards around identifying patients and doing some planning with them around their future health care [Clinician 5] |
|  |  | So we've used the opportunity to work more closely with the doctors around advanced care planning, which will work well into the future as it's now a big expectation of health care accreditors that this has to happen [Clinician 5] |
|  | COVID-19 | Inpatient numbers reduced by 10-20% as everyone socially isolated. But the numbers are picking up again now as people with chronic illness are finding that their illness has progressed [Clinician 2] |
| **Other** |  |  |
|  | Role of other health professionals | I think social work is going to be a big thing to support the advanced care planning. In terms of do we do this high-risk thing or can we should we send them home with some support at home for their remaining time? And what do they want? [Auditor 2] |
|  |  | It should flag to the allied health staff, you know, that they're frail and so on, maybe they won't push them quite so hard to get up and going and they need to have a chat to the consultants [Clinician 3] |
|  | Value of research opportunities | So I'm still a nurse, but my goal is to find another position that's similar to what InterACT has offered, because I've just really enjoyed this particular role [Auditor 2] |
|  |  | it really helps with trying to get us involved in more research, you know, in the future. So it's really to me, it's a really good opportunity for us to start building some, you know, links to the Uni and getting involved where we can because it works well [Clinician 5] |
|  | Transferability to other cohorts and sustainability | I'd be intrigued to see how the results pan out because, you know, I guess in our world, what we also meet is a bunch of people, way way under 75, who, in fact, probably have similar levels of frailty, debility and poor prognosis, and so I'd be interested to know whether this could sort of be translatable to an age group that perhaps wasn't yet captured by the design of the tools and the design, because we see that, I guess, mostly in the cancer population, but in things like motor neurone disease, sometimes it's people that are decades younger than would have ever been screened for through the InterACT study [Clinician 6] |
|  |  | In terms of if there was such a position created, like, how would that work? How many hours or FTE people would you really need across a hospital this size? [Clinician 6] |
|  |  | Would be so good if there was something that would just calculate as they came through emergency. Yeah, that would be great and automatically kind of flag them as at risk somehow, yeah, that's a whole other kettle of fish [Auditor 1] |
|  |  | It's going to be really difficult because no one will be doing the score, so they need to work out an algorithm that develops the score out of the information that's already provided in the various data sets. So we already have like a frailty score and and, you know, the Bartels or whatever, you know, the various things that the nurses put into the data anyway so if we could get the algorithm pulled out of that, which gives us the score then that would mean that it would be ongoing. But if we now have to get a dedicated SPICT nurse or a dedicated CRisTAL nurse, that's going to be difficult [Clinician 3] |

**Table S4.** Summary of Hospital Y interview data using the Consolidated Framework for Implementation Research (CFIR)

| **CFIR domain** | **Construct** | **Quote/Findings** |
| --- | --- | --- |
| **Intervention Characteristics** |  |  |
|  | Relative Advantage | (previous studies) tackled at a nursing level and we know that’s not the whole picture because the medical staff have not been involved and they’re the ones that ultimately make some of these decisions. So, I think what was really great was that it targeted the medical leads [Executive Advisory Group 3] |
|  | Complexity |  |
|  | Evidence strength and quality | One of their cardiologists came back and said they didn't like the science around this and disputed a lot of it and I think name got involved in trying to get them to understand it wasn't about changing the care [Executive Advisory Group 3] |
|  |  | There's a geriatrician who believes that we're being ageist and has done a bit of a bit of research and is very vocal about that [Research Team 1] |
|  |  | I’m a great believer in the SPICT tools and I’ve done my own research in this area too. It’s pretty sensitive, but it’s also pretty specific too [Executive Advisory Group 3] |
|  | Design quality and packaging | I was under the impression that we could have the same thing with a visual flag. But we weren’t having a visual code we were having a referral and referral basically doesn’t go anywhere [Research Team 1] |
| **Inner Setting** |  |  |
|  | Available resources | Auditors came and went because of maternity leave, other secondments and so forth and you can’t help that [Research Team 1] |
|  | Culture | there’s not a can-do attitude there at all, everything we asked or wanted to do was difficult, it’s probably organisational culture [Research Team 2] |
|  |  | Engaging the clinical teams, that was, they’re just different. They’re different than Site 1. Site 1 is very, very engaging, you’d get a reply to your email [Research Team 2] |
|  |  | cultural changes need to occur over time [Clinician 10] |
|  | Compatibility | But we generally had identified our patients so the InterACT study criteria didn't necessarily achieve anything different out of our patient cohort [Clinician 7] |
|  |  | We didn't feel that this study, especially using the CRiSTAL and the SPICT tools, were going to tell us anything different. But as cardiologists, we feel we do a pretty decent job in identifying futility [Clinician 7] |
|  |  | we’ve already identified those patients before you do, before the trial has identified them [Clinician 7] |
|  |  | Every single one of our admissions is unplanned [Clinician 10] |
|  |  | I’m not sure the screening tools work well for stroke [Clinician 10] |
|  |  | One of the medical officers might be reading the chart and then I’d be like oh can I have that once you’re with that please? Yeah no problems, who are you? And you’d be like oh I’m with the InterACT study…what’s the InterACT study? I think that’s got to do with the communication from the consultant [Auditor 3] |
|  |  | there was always a lot of debate over whose responsibility advanced care planning was, however no one actually put up their hand to say this is like the facilitator’s role or this is social workers, medical officers or whatever it was, so I think it was a big higgledy piggledy to begin with [Auditor 3] |
|  | Relative priority | We found it didn't really help or add anything to our clinical care was because we have a bed parliament with all the senior clinicians and their registrars, senior nursing staff meet every Monday, Wednesday, Friday at seven thirty [Clinician 7] |
|  |  | It has been a real eye opener that you’ve got all these red flags and no one’s having these conversations with these people it’s like why? Why are people not even referring them or doing something [Auditor 3] |
|  |  | you’re not dead until you’ve had your sixth bone marrow transplant and your 14th trip to the intensive care. That kind of hope at all costs every possible intervention [Executive Advisory Group 3] |
| **Characteristics of individuals** |  |  |
|  | Self-efficacy | I think people that struggle with end-of-life discussions are people who don’t really have great communication skills to begin with [Executive Advisory Group 2] |
|  |  | I think there’s a significant difference between one clinician and the next in their capacity to genuinely engage with patients and their families [Executive Advisory Group 2] |
|  |  | A lot of people find it really difficulty to have those conversations, they don’t either have the conversation skills to do it or they feel it’s one of those topics that can be a little bit sensitive [Auditor 3] |
|  |  | Documentation was very poor when it came to this, very poor, some like a very small percentage where I would say, oh you want to take a photo of this one because it looks so good, oh it’s even dated [Auditor 3] |
|  |  | Documentation is failing abysmally. You’d read through doctor’s notes and in the plan, it’d just be like all their numbers of what they’re doing and then ACP or ARP so it’s like that’s nice, but did you actually have a conversation with the patient? You’d go to the ARP to see, maybe, just maybe they put the discussion like a bit of detail, maybe one or two sentences on the ARP and it would be blank [Auditor 3] |
|  | Knowledge and beliefs | We were concerned that that may be used to (number of positive patients), you know, cast the department in sort of a negative light [Clinician 7] |
|  |  | I’m not confident at Site 2 that the majority of consultants in the big teams actually know what the project is or what the expectation is [Research Team 1] |
|  |  | I feel like there’s been a lot of lack of communication on the medical team side of things with regards to actually informing their staff members about this study. Even now we’re in the intervention phase I will still come across the occasional medical officer that’ll be like what’s that? [Auditor 3] |
|  |  | I don’t think people have that as high as a priority until they actually potentially either involved in a situation themselves or with a family member or a loved one where they’re put in a situation like that, and they like actually think this is really important, this is something that we should be putting a bigger focus on that [Auditor 3] |
|  |  | We have a lot of older patients and we often come up against the situation where we need to consider whether we intervene or how much we do in terms of investigation [Clinician 9] |
|  |  | Everyone when I’ve explained to them, has always been very much, that sounds like a good idea [Auditor 3] |
|  |  | I do think there are some clinicians that have got this really, really strong sanctity of life thing [Executive Advisory Group 2] |
|  |  | There's not a lot of focus on what a person's values and wishes and beliefs and preferences is. It's really all about the medical science side of things and what we can and can't do for you compared to actually getting that little bit of shared decision making out there and involving a person in their in their care [Auditor 3] |
|  |  | Essentially, we're a cure all facility, the paradigm is around cure. Having said that, we have very strong engagement across all the services with palliative care. Yeah, those principles are very well embedded [Executive Advisory Group 3] |
| **Implementation Process** |  |  |
|  | Engaging key stakeholders | It doesn’t feel as if the interest is there from the executive advisory group. Every time we go to try and have a meeting, we end up with like two people, we certainly can’t get the key people there [Research Team 2] |
|  |  | I think if you don't have senior medical leadership, you don't get leverage of things put forward. I think the nurses are fantastic because they're the ones that are there every day they see what's going on with the patients and they want to see more happen. But you've got to get buy in from senior medical to actually drive the team response and to listen to the nurses [Executive Advisory Group 3] |
|  |  | I did have to do a lot of work to get *name* on board as well, took a little while. So then once we got the executive advisory group sort of these nominees ready and then to pull them together again, *name* was really good in auspicing (supporting) [Research Team 2] |
|  |  | The big teams so Cardiology, cancelled on me like four times in a row and then basically met and the director said, I basically don't have time for this don't send me an email I cannot reply to you, my home computer has just died, I have no capacity. And I was like... okay, is there someone else I can send something to? [Research Team 1] |
|  |  | Then when there wasn’t that drive from up top really supporting it to ensure that there would be some form of sustainability across the whole facility, it fell out of sight [Auditor 3] |
|  | Internal appointed implementation | we had the site lead, we had her right from the start and so she was a great sort of ally with me [Research Team 1] |
|  |  | When we came back from lock down, the other auditor was pregnant, so instead of having a 12 month contract, she got a three month contract and she couldn't start straight away, and it just so then we had a fill in and then we she left and the fill in stayed and she needed a lot of support, and then she just left she took a job [Research Team 1] |
|  | External change agent |  |
|  |  | It was a QUT funded project, we had Chris, who was very much hands on whenever we needed her, she was always able to be contacted and also we were very lucky that we sat with the research centre at site 2 and therefore we were really supported from that side of things. Because with some projects, even though they’re run by outside affiliations and you’re a guest going into that facility, you can sometimes feel like an intruder [Auditor 3] |
|  | Opinion Leaders | We used *name* a lot and we sort of had to use her and probably had to use her in a sort of almost like bringing in the artillery manner [Research Team 1] |
|  |  | You’ve got to get buy in from senior medical to actually drive the team response [Research Team 1] |
|  |  | She does have a lot of weight at the hospital. So thank goodness I don't know what we would have done if we hadn't had her, you know it would have been a really difficult hospital to get into [Research Team 1] |
|  | Executing |  |
|  |  | A director who had signed off on something and probably hadn’t communicated completely to other senior clinicians about what it means [Executive Advisory Group 3] |
|  |  | in hindsight, my learning would be we should have never had I would never, ever do, and in such a big in kind commitment from a hospital, it just doesn't work [Research Team 2] |
|  |  | The difficulties have included that the hospital didn't honor its commitments around in-kind support. Yeah, there was about two years of difficulties with actually getting the contractual agreements set up. Nothing to do with anything to do with the content of the project. Yeah. It's just got everything to do with running research in an institution like this. And it's not different to any other partnership grant that we have where someone agrees to it and then those people have shifted on by the time the grant gets up and then no one wants to honour the project and the budget situation [Executive Advisory Group 2] |
|  | Champion | So she didn’t have a lot of in at Hospital Y, she wasn’t able to access their system, and people wondered who she was [Research Team 1] |
| **Other** |  |  |
|  | Value of research and pre-existing partnerships | Set up a kind of collaborative research agreement, which is where we did those sort of chart audits and some preliminary interviews and I set up a monthly meeting from 2010 or early 2011 onwards and we met every single month until probably about 2015. And that resulted in the ARC partnership grant, it resulted in all of the papers that have been written that inform this particular project were all come out of that [Executive Advisory Group 2] |
|  |  | The research I did showed that sixty seven per cent of people actually died a year within the year. We use SPICT criteria on a one-day study. Now that tells me that those people needed to have certain things put in place. So, you know, we're not dealing we are dealing with a population of people that we need to address the relevance of certain clinical interventions [Executive Advisory Group 3] |
|  | Transferability to other cohorts | I hope they have in their analysis for some prespecified plan to review subgroups of patients differently to stroke patients, many patients in whom the presenting illness is the most strong predictor of outcome. It's like trauma, kind of depends on what it is, what was the acute reason remission is, as opposed to, as you say, a patient who's got a collection of co-morbidities and some chronic unwellness who has become more or less destabilized by the presentation. Those are the kind of patients I think it would benefit the most from this, tool, because when people say yes, just another patient with this mild to moderate illness and don't realize what the background is [Clinician 10] |

**Table S5.** Summary of Hospital Z interview data using the Consolidated Framework for Implementation Research (CFIR)

| **CFIR domain** | **Construct** | **Quote/Findings** |
| --- | --- | --- |
| **Intervention Characteristics** |  |  |
|  | Relative Advantage | There are multiple reasons for non beneficial care being provided, and the initial article based on which you have designed this study has shown at multiple levels there are barriers and I see this intervention addresses some of them, not all of the barriers [Clinician 12] |
|  | Complexity | I think that the study wasn't bad, I think that it was well on the methodology [Executive Advisory Group 6] |
|  | Design quality and packaging | I am familiar with the SPICT more than the CRiSTAL tool, and I guess it has raised names of patients that I certainly weren't aware of [Clinician 13] |
| **Inner Setting** |  |  |
|  | Readiness for implementation | They suffered by being the longest to switch over. We did get things like: what’s this study about [Research Team 1] |
|  | Available resources | Being closer to the engine room at Hospital Z. We’re very linked with service delivery centres at the other hospitals, we feel like we’ve probably got more access into the quality and governance sort of teams [Research Team 2] |
|  |  | The biggest problem, was finding internal research staff who could provide time, who could access the data systems. So you have to be a designated person of Queensland Health to access the actual system IEMR. And we don't have a lot of capacity in terms of full time equivalent of research to allow that to happen. [Executive Advisory Group 4] |
|  | Culture | Clinicians may come in, treat a patient, and leave without building an ongoing relationship, often only seeing patients for a procedure [Research Team 1] |
|  |  | Pushing towards developing the research culture, which had previously been less advanced, less sort of inbuilt, ingrained [Research Team 1] |
|  |  | there’s a lot of bureaucracy, a lot of red tape, a lot of paperwork to be done, so we don’t even bother [Clinician 14] |
|  |  | I already know it's pretty much gone back to what it was because that's what happens, the team just reverts because the culture doesn't change that quickly [Executive Advisory Group 5] |
|  | Access to knowledge and information | that the idea of research is probably not, as you know, ingrained in their practice as it is at the other hospitals [Research Team 1] |
|  | Relative priority | the amount of money and I know this sounds really harsh, but we waste potentially when we could be just making people’s end-of-life much more comfortable or a smoother transition for them [Clinician 15] |
|  |  | The lack of funding and agenda to grow empires so that making hospitals bigger, getting more and more hospital-based staff, not about stopping people going to hospital [Executive Advisory Group 5] |
|  |  | We’ve had no new hospital avoidance money funded in the *location* for more than five years. The only thing we have are actually budget cuts [Executive Advisory Group 5] |
|  |  | they’ve got an interest because they do have those patients for a very long time, whereas in cardiology and respiratory, they tend to have much more of a treatment-based system where, you know, you’re either with them or you’re with general medicine [Clinician 13] |
|  | Networks and communication | External access of iEMR was required at $22/month for auditing purposes [Research Team 1] |
|  | Access to knowledge and information | unless they were on ward service and they were seeing these patients, it may not have stayed on their radar [Clinician 13] |
| **Characteristics of individuals** |  |  |
|  | Self-efficacy | They don't have the time or the willingness [Research Team 1] |
|  |  | I was never trained to do this, I don’t have the expertise to do this, I don’t think it’s my job because my job as a doctor is to cure and to death deny. It’s against God’s will [Executive Advisory Group 5] |
|  |  | You’re either good at it or you’re not as a clinician [Executive Advisory Group 6] |
|  |  | it’s not that people don’t recognise it, even if they do recognise it, they may not have the skillset necessary, and resources necessary to act properly [Clinician 12] |
|  |  | Apart from oncology and palliative care they (clinicians) don’t receive any structured communication training. Palliative care is the only speciality where interested people will receive communication training [Clinician 12] |
|  |  | Left too late and the relative is about to die in the medical ward then the GP’s and emergency department palm it to us. We the physicians then have to do it in a busy medical decision unit with the junior doctors who are not the best in communication skills [Clinician 14] |
|  | Knowledge and beliefs |  |
|  |  | it’s very valuable, it is better so they’re doing it and palliating the patients more early or they’re withdrawing the treatment more early [Clinician 14] |
|  |  | Medically, legally, it's in our best interest, not only the patient to have the advance care planning done on day zero, to have the ARP done on day zero so that the patient's journey is easy for them and for us. So now that we know all these things are happening now, we are more aware that this should be thought about and it should be done, and it should not just be laid by the wayside [Clinician 14] |
|  |  | to show that these (advanced care planning) are no brainers [Executive Advisory Group 5] |
|  |  | I’m very thankful you got me involved, so happy to be involved with this [Clinician 14] |
|  |  | looking at what else we can do and what is effective in terms of alerting clinicians to inappropriate or ineffective care [Clinician 13] |
|  |  | remind a busy clinician you have a seriously ill patient, have you thought of if the intervention you’re offering is going to benefit this patient or not, that’s sort of a reminder and it does help people who have the right attitude if they have forgotten in the midst of the busy work [Clinician 14] |
|  | Culture | Culture change doesn’t occur when you put somebody in for 12 months. Culture change requires years, and it requires resourcing to put the culture change in and then once it’s there, the hardest thing is to keep the change there, otherwise it’s just an effort to revert to the norm. We all know what the easiest way is with end-of-life care that has to be: death denying, there’s to overtreat, and it is to not communicate that people need something different. It is far easier to send somebody to the ED and to spend five thousand dollars in ED, two thousand dollars a day in the next department, in the next department, then to have that discussion [Executive Advisory Group 5] |
|  |  | I sort of sense overall that the project didn’t heighten their (clinicians) awareness of actively change their thinking around inappropriate or burdensome treatment [Clinician 13] |
|  |  | Junior doctors are quite happy to be involved because of experience level, some of them are very experienced and can do it, some are not so experienced, so they want to watch us do it and learn [Clinician 14] |
| **Implementation Process** |  |  |
|  | Engaging key stakeholders | Really receptive, I’m surprised that they managed to get the executive so engaged [Executive Advisory Group 4] |
|  |  | I think they way it met was good and then coming together when it was needing to come together. So, it was really good the way we met, it was to try and just move things forward or a communication update which was really important as well [Executive Advisory Group 6] |
|  |  | Certainly our executive director of Clinical Governance, Research and education has been very supportive. I've heard that others also supportive, I've seen it personally. [Clinician 12] |
|  | Formally Appointed Internal Implementation Leaders | it was really difficult to get a site lead there, they were very hard, had to do lots of back and forth negotiating, you know, reinforcing contracts. But we did a lot of work, got that person [Research Team 1] |
|  |  | It was hard to get the inroads in the right person [Research Team 2] |
|  | External change agent | quite good, they have been in touch with us for quite a while, they were quite happy to come in and do a presentation at the unit level. They have been quite accessible and available for the team [Clinician 12] |
|  |  | they’re very helpful and respond to queries immediately. It’s been pretty good working with them [Auditor 5] |
|  |  | QUT’s interaction with health service affiliation, so they understood the health system probably more compared to other people from academic institutions that we’ve had to work with [Executive Advisory Group 4] |
|  | Champions | Routinely mentioning InterACT at every high-level governance meeting [Research Team 2] |
|  | Planning | Extremely well-designed study [Clinician 14] |
|  |  | I'd say the problem we have is probably the same as every public health system, the different the legislation and the processes that public health can be quite restrictive, particularly when we want to engage with external partners and run projects like this. Yes, so we've had to be quite innovative and think outside the square how we can get things to happen. [Executive Advisory Group 4] |
|  | Executing | because our hospital has the IEMR, we mainly extract everything on the computer. We don't ever actually go on site to actually look for the information we need, which is very, very efficient in a way [Auditor 4] |
| **Outer Setting** |  |  |
|  | Needs and resources | Patients are very happy and very open to these conversations [Clinician 14] |
|  | External policy and incentives | end-of-life care, palliative care, advanced care planning are not funded appropriately and it’s very clear in Queensland [Executive Advisory Group 5] |
|  |  | activity-based funding (ABF) only encourages overtreatment [Executive Advisory Group 5] |
|  |  | advanced care planning has gone through the floor in every health service because there is no money with it so the health service under ABF just goes to the next thing that brings in money but it’s repeated policy failures by thinking short term [Executive Advisory Group 5] |
|  |  | Sometimes relatives just don’t want us to give up, even though we know the patient is dying, and sometimes we are compelled to do more because relatives have a lot of power here in Queensland because of the legislation [Clinician 14] |
|  |  | The only thing that I potentially think that started a lot of people thinking is that movement to the assisted dying legislation that came through in the last year that's been pushed sort of for the last six months or so. So I don't know if that's just brought people more aware of end-of-life matters, and that's part of it but that would be the only tie in that I could potentially see [Executive Advisory Group 6] |
|  |  | Ryan's rule was originally designed for missed diagnoses in emergencies where the wrong decision has been made and relatives are concerned about their loved ones in hospital, but the system is being used in the wrong way [Clinician 14] |
|  |  | Pretty much every month we have one or two Ryan's rules, often there's just disagreement and lack of communication, lack of understanding on the relative’s part. Sometimes they force us to do full resuscitation, and someone is very frail, elderly and dying, and you give the best explanation as to why resuscitation is futile, but they don't want to understand or whatever reason, they don't want to listen to us or don't want to believe or they are in denial that their relative is dying [Clinician 14] |
| **Other** | Economic Research Impact | Ultimately this was a I think it was an MRFF funded project, it was only for a certain time as it is. So I think that would need to be and I don't know if they've done that, but an economic analysis to show these are no brainers [Executive Advisory Group 5] |
|  |  | it would be fantastic if we could go one step further with it where they actually realized that, you know, understand the economic you know, the economics of actually, you know, treating people that are at end of life and the amount of money that and I know this sounds really harsh, but we waste potentially we could be just making people's end of life much more comfortable or a much smoother transition for them [Clinician 15] |
|  | Sustainability | It's highly unlikely unless we put a process in place where I think there can be regular reminders, and it's my feeling that as long as the process is manual, it will be hard [Clinician 12] |
|  |  | I think it's just a matter of working smarter, particularly if you've got the IT processes in place. It's just about engaging with IT or other relevant people to work a little bit smarter [Clinician 13] |

**S6:** Example Interview Guide

## **Interview Outline: Phase 2 Implementation**

#### Aim

Gain insight into participants’ experience of implementing the InterACT prospective feedback loop

intervention, including:

1. the process of receiving screened patient review data
2. implementing clinical response pathways in response to the review data
3. impact on providing patient care
4. extent and fidelity of implementation.

#### Structure

The structure of the discussion groups and interviews will be based on the Consolidated Framework for Implementation Research (CFIR) domains and constructs. Questions will be tailored to reflect the timing of the discussion groups and interviews in relation to the trial implementation phases.

#### Content

Review aims of session, participant information sheet, particularly confidentiality, consent, privacy and right not to participate. Collect signed consent forms.

***Note: not all sections may be covered in each session, depending on participant numbers, roles, flow of discussion, time constraints and particular trial phase.***

|  | |
| --- | --- |
| **Domain** | **Question content topics** |
| **INTERVENTION CHARACTERISTICS** | Perceptions of the intervention evidence and need for change  The degree to which the intervention is/was adaptable to meet local needs. Extent of ease of use of the intervention |
| **INNER SETTING** | Implementation climate:  Perceived need for change  Fit and alignment of the intervention with practice and workflows |
|  | Readiness for implementation: Leadership engagement Organisational support  Access to knowledge and information |
| **CHARACTERISTICS OF INDIVIDUALS** | Attitudes and values about the intervention  Self-efficacy and ability to implement the intervention |
| **PROCESS** | Extent and impact of planning for implementation  Role and impact of opinion leaders, implementation leaders, champions, change agents and stakeholders Response to the intervention |
|  | Execution:  How is/was it working? What could be/have been improved? Reflection and evaluation:  Progress and quality of implementation |

## **Interview Outline: Phase 3 Post-implementation**

#### Aim

Gain insight into participants’ experience of implementing the InterACT prospective feedback loop

intervention, including:

- 1. the process of receiving screened patient review data
  2. implementing clinical response pathways in response to the review data
  3. impact on providing patient care
  4. extent and fidelity of implementation.

#### Structure

The structure of the discussion groups and interviews will be based on the Consolidated Framework for Implementation Research (CFIR) domains and constructs. Questions will be tailored to reflect the timing of the discussion groups and interviews in relation to the trial implementation phases.

#### Content

Review aims of session, participant information sheet, particularly confidentiality, consent, privacy and right not to participate. Collect signed consent forms.

***Note: not all sections may be covered in each session, depending on participant numbers, roles, flow of discussion, time constraints and particular trial phase.***

|  | |
| --- | --- |
| **Domain** | **Question content topics** |
| **INTERVENTION CHARACTERISTICS** | Perceptions of the intervention evidence and need for change  The degree to which the intervention is/was adaptable to meet local needs. Extent of ease of use of the intervention |
| **OUTER SETTING** | External barriers and facilitators to meeting patient needs Nature and impact of external policy and incentives  External impetus to change practice. |
| **INNER SETTING** | Implementation climate:  Perceived need for change  Fit and alignment of the intervention with practice and workflows |
|  | Readiness for implementation: Leadership engagement Organisational support  Access to knowledge and information |
| **CHARACTERISTICS OF INDIVIDUALS** | Attitudes and values about the intervention  Self-efficacy and ability to implement the intervention |
| **PROCESS** | Extent and impact of planning for implementation  Role and impact of opinion leaders, implementation leaders, champions, change agents and stakeholders  Response to the intervention |
|  | Execution:  How is/was it working? What could be/have been improved? Reflection and evaluation:  Progress and quality of implementation |
